# DRESS Syndrome due to Nevirapine Treated with Methylprednisolone

**DOI:** 10.1155/2013/269501

**Published:** 2013-08-01

**Authors:** Henrique Pott Junior, Gisele Cristina Gosuen, Ana Cristina Gales

**Affiliations:** Division of Infectious Diseases, Federal University of São Paulo (UNIFESP), São Paulo, SP, Brazil

## Abstract

Nevirapine-induced DRESS syndrome is uncommon but a potentially life-threatening condition, with significant morbidity and mortality rates due to multiple-organ involvement. The authors report a case of a 47-year-old HIV-infected female patient who presented with fever, right hypochondrium pain, jaundice, and skin rash. The Nevirapine-induced DRESS syndrome was suspected based on clinical presentation and RegiSCAR scoring system. This case highlights the need for a high index of clinical suspicion among HIV-infected patients with severe skin eruption and systemic symptoms.

## 1. Introduction

The Drug Reaction with Eosinophilia and Systemic Symptom (DRESS) is a severe idiosyncratic adverse drug reaction with multiple-organ involvement, which was first described by Bocquet et al. in 1996 and has been reported mostly in association with aromatic anticonvulsants, sulfonamides, allopurinol, and Nevirapine [1, 2].

 The acronym designated by Bocquet et al. [1] describes the clinical manifestations of a potentially life-threatening syndrome that includes severe skin eruption, fever, hematologic abnormalities (eosinophilia or atypical lymphocytes), and internal organ dysfunction. The other noteworthy features are a delayed onset, usually 2–6 weeks after the initiation of drug therapy, and the possible persistence or aggravation of symptoms despite the discontinuation of the culprit drug. The mortality rate has been estimated at around 10%, and fatal outcome is most often associated with liver failure.

We describe a 47-year-old woman infected with HIV whose chronology of signs and symptoms and RegiSCAR score suggest a case of adverse drug reaction associated with Nevirapine compatible with diagnosis of DRESS syndrome. She was successfully treated with intravenous methylprednisolone.

## 2. Case Report

A 47-year-old woman with human immunodeficiency virus 1 (HIV-1) infection was admitted to the São Paulo Hospital because of fever, right hypochondrium pain, jaundice, and skin rash. In the emergency department, the patient reported fever (temperature, 39°C), myalgias and arthralgias, choluria, pruritus, and gradual onset of right hypochondrium pain. The patient had been well until twenty days before admission, when a diffuse maculopapular rash accompanied by discrete yellow discoloration of the conjunctive of the eyes developed, followed by a myalgias and arthralgias, choluria, pruritus, and vague pain in the abdomen and lower back. Three days before admission, nausea and vomiting developed, and oral intake decreased.

The HIV-1 infection was detected on a routine testing three years earlier. The CD4 T-lymphocyte count was 436 cells per cubic millimeter, and the HIV viral load was not available. There was no evidence of active coinfection with hepatitis B or C, syphilis, *Mycobacterium tuberculosis*, or history of opportunistic infections. Positive IgG but negative IgM antibodies to cytomegalovirus (CMV) and *Toxoplasma gondii* were detected in the patient's serum. After the diagnosis of HIV-1 infection, it was started with Zidovudine, Lamivudine, and Lopinavir boosted with Ritonavir; subsequently, CD4 T-cell counts remained consistently above 500 cells per cubic millimeter, and there was a fall in the plasma HIV load to less than 50 RNA copies per milliliter. One year before admission, daily gastrointestinal intolerance developed, which gradually worsened. Forty-five days before admission the patient was admitted to her reference outpatient care clinic, when the medical assistant changed the antiretroviral therapy to Nevirapine, Zidovudine, and Lamivudine. She was admitted to our hospital twenty days after initiating the new regimen because of a diffuse maculopapular rash accompanied by discrete yellow discoloration of the whites of the eyes that has evolved in the past ten days due to intensification of the yellow discoloration of the skin and mucous membranes, fever (temperature, 39°C), myalgias and arthralgias, choluria, pruritus, and gradual onset of right hypochondrium pain. She did not take other forms of medication.

On examination, the patient was awake, alert, and oriented. The blood pressure was 95/66 mm Hg; other vital signs were normal. There was marked conjunctival and skin icterus, and the skin was yellow, with diffuse maculopapular rash but without spider angiomas, palmar erythema, or telangiectasias. There was no anathema in her oral cavity nor lymph nodes enlargement. The abdomen was soft, with tenderness on right hypochondrium, without distention or organomegaly. Neurologic examination revealed normal speech and attention and no asterixis. The remaining of the physical examination was unremarkable.

Results of a complete blood count and differential count were normal as were serum levels of glucose, calcium, phosphorus, magnesium, total protein, albumin, globulin, amylase, and lipase. There was important elevation of the liver enzymes combined with elevation of bilirubins, alkaline phosphatase, and gamma-glutamyl transpeptidase. Also, according to the RIFLE criteria, there was renal failure determined by a threefold increase in the serum creatinine combined with decrease of glomerular filtration rate by 75 percent. Testing was negative for cytomegalovirus (CMV) antigenemia, antibodies to HBV and HCV, and IgM antibodies to hepatitis A virus (HAV); other test results are shown in Table 1. An electrocardiogram was normal. Ultrasonography of the abdomen revealed liver presenting regular shape and size with homogeneously increased echogenicity of liver parenchyma. Skin biopsy and its histopathologic analysis revealed interface dermatitis with mixed perivascular infiltrate suggesting a drug reaction pattern.

Based on a RegiSCAR's scoring system of 5 points, a probable diagnosis of Nevirapine-induced DRESS syndrome was made. The rapid development of hepatocellular and renal dysfunction determined severity to this case, and the patient was admitted to a semi-intensive care unit. All antiretroviral treatment use was discontinued, and methylprednisolone 1 mg/kg/day was started. Laboratory followup was also shown in Table 1. After 20 days of hospitalization, the patient recovered sufficiently to be safely discharged from hospital. Patient was given instruction to schedule an outpatient clinic consultation for early return (7 days) and followup. 

## 3. Discussion

Nevirapine is an oral medication that is used in combination with other antiretroviral agents for the treatment of human immunodeficiency virus disease. It belongs to a class of drugs known as nonnucleoside reverse transcriptase inhibitors (a “nonnuke” or NNRTI). The drug exerts a virustatic effect by acting as a specific, noncompetitive HIV-1 reverse transcriptase (RT) inhibitor. The drug binds directly to heterodimeric HIV-1 RT and inhibits the RT activity by disrupting the catalytic site of the enzyme [3].

 To establish its safety profile, Pollard et al. conducted a review of data from prospective US and international clinical trials involving a total of 906 adult patients and 468 pediatric patients treated with NVP. Drug-related adverse events were similar in adults and children, with rash and nausea most frequently reported in adults. A separate analysis of rash based on data from adult patients in controlled trials demonstrated a 16% rate of NVP-attributable rash in these patients. Of patients with NVP-associated rash, 65% developed rash within the first 6 weeks of therapy. No cases of DRESS syndrome were reported [3].

 Yet this drug is associated with a drug hypersensitivity syndrome in approximately 5% of those starting the drug and Stevens-Johnson syndrome/toxic epidermal necrolysis in 0.3% or less of those initiating the drug [4]. DRESS syndrome has been initially described as the anticonvulsant hypersensitivity syndrome [5, 6] even though recently many studies have reported an increased association of DRESS syndrome with Nevirapine, becoming the third leading cause [5–11]. Diagnosis can be difficult due to the variable presentation of the syndrome and is more often obtained by exclusion.

In an effort to define more accurately the DRESS syndrome, a scoring system has been recently developed: the RegiSCAR scoring system [5, 6]. RegiSCAR constitutes a European registry of severe cutaneous adverse reaction (SCAR), including Stevens-Johnson syndrome, toxic epidermal necrolysis, acute generalized exanthematous pustulosis, and DRESS. One of the aims of this registry is to delineate each of these SCARs as distinct entities. In this line, the RegiSCAR's scoring system has been designed to grade DRESS cases as “no,” “possible,” “probable,” or “definite” case [5, 6]. With this scoring system, our case could be classified as probable DRESS scoring 5 points: fever ≥38.5°C (1 point), eosinophilia (0 points), skin rash >50% body surface area (1 point), biopsy suggesting DRESS (0 points), organ involvement (liver and kidney—2 points), resolution ≥15 days (0 points), and negative evaluation of other potential causes (1 point). Approximately at the 10th day of hospital admission 1% (115) of atypical lymphocytes were observed in peripheral blood counts, which could establish a definite case of DRESS scoring 6 at RegiSCAR. There were no lymph nodes enlargements.

Recently, Wongkitisophon et al. [12] reviewed the clinical presentations, laboratory and pathological findings, and prognosis of all patients with DRESS, diagnosed from January 2005 to April 2010 in a tertiary centre in Thailand. The most common culprits medications were phenytoin, allopurinol, and Nevirapine. Mean duration of drug administration before the onset of symptoms was 34 days. The latent period was longer for allopurinol (103 days) and shorter for Nevirapine (10 days). Skin rash was seen in all patients, while fever and lymphadenopathy were found in 88.9% and 22.2%, respectively. Hepatic and hematological involvements were the two most common systemic complications, occurring in 96.3% and 85.2%, respectively [12]. Regarding clinical features, our case had similar profile and evolution, despite the absence of lymph nodes involvement.

When available, molecular typing methods and the ability to analyze the whole genome in an unbiased fashion can lead to some remarkable findings of the role of the human leukocyte antigen (HLA) genes as genomic biomarkers of predisposition. Recently, HIV-positive Chinese patients treated with a Nevirapine-based regimen were investigated for the association between Nevirapine hypersensitivity reaction and HLA allele. More HLA-Cw*04 alleles were observed in Nevirapine hypersensitivity reaction cases than in Nevirapine-tolerant cases (*P* = 0.029). Nevertheless, Umapathy et al. [13] revealed a highly significant association of HLA B35 (OR: 3.378; *P* value 0.0032) with Nevirapine-induced skin rash. Further, significant HLA B7 (OR: 0.292; *P* value 0.0085), HLA B8 (OR: 0.272; *P* value 0.0825), and HLA B15 (OR: 0.272; *P* value 0.0825) were also found to be decreased significantly among the Nevirapine-hypersensitive patients. Further investigations are needed to understand whether these HLA alleles are truly causative or acting as surrogate markers of predisposition to Nevirapine hypersensitivity reaction.

Yet another important aspect of the follow-up process is the treatment. Until now, there is no consensus on the indication of treatments with corticosteroids or corticosteroids with/out intravenous immunoglobulin besides clinical support. Bourezane et al. described the first case of hypersensitivity syndrome (HSS) associated with Nevirapine therapy who was successfully treated with intravenous methylprednisolone [7]. Lanzafame et al. reported the first case of DRESS syndrome complicated by meningoencephalitis associated with Nevirapine therapy [8]. Claudio et al. reported a 31-year-old man diagnosed as having HIV and was following an antiretroviral treatment with high viral load, which developed Nevirapine hypersensitivity syndrome after changing antiretroviral treatment regimens. Nevirapine therapy was discontinued when symptoms appeared no particular treatment was started, and 7 days later the patient had no symptoms, and laboratory test findings were normalized [9]. Fields et al. described a patient with HIV who developed Nevirapine hypersensitivity syndrome who was successfully treated with intravenous immune globulin [10]. Santos et al. described a 12-year-old girl infected with human immunodeficiency virus who developed hypersensitivity to Nevirapine therapy. She was successfully treated with intravenous immune globulin [11]. And, according to Wongkitisophon et al.'s recent review, most patients with Nevirapine-associated DRESS syndrome are treated with systemic corticosteroids, either intravenous dexamethasone (15–20 mg/day) or oral prednisolone (0.5–0.7 mg/kg/day). Overall, the mean duration of treatment is 49 days [12]. Considering the absence of first-line therapy consensus, we decided to treat our patient with intravenous methylprednisolone (1 mg/kg/day) during 20 days. The patient had a good response to treatment evolving with a progressive decrease in markers of liver injury, recovery of renal function, and disappearance of systemic symptoms.

Nevertheless, based on hepatotoxicity observed in controlled and uncontrolled studies, now it is established in the literature that Nevirapine, including extended-release Nevirapine, should not be initiated in adult females with CD4 counts greater than 250 cells/mm^3^ or in adult males with CD4 counts greater than 400 cells/mm^3^ unless the benefit outweighs the risk [14, 15]. This was the cause of the subsequent events presented by our patient. Also, considering Nevirapine specific pharmacokinetic properties, the impairment of renal function by Nevirapine-induced acute interstitial nephritis resulted in reduction of its renal clearance maintaining the allergenic stimulus. Breining et al. reported a similar case in which an immunocompetent woman treated with Nevirapine for postexposure HIV prophylaxis maintained an effective allergenic stimulus during a period of 12 days after interruption of treatment [16].

## 4. Conclusion

In conclusion, DRESS is potentially life-threatening, with significant morbidity and estimated mortality of 10% [2]. Our case shows a case of Nevirapine-induced DRESS syndrome, complicated by acute hepatitis and interstitial nephritis, treated with intravenous methylprednisolone, and followed by successful outcome. We believe that early detection, discontinuation of the suspected drug, and administration of systemic corticosteroids are the key steps in management of DRESS.

## Figures and Tables

**Table 1:** Evolution of laboratory test results during hospitalization.

Hematologic and blood chemical laboratory test results*					
	Hospital admission	10-day Hospital admission	20-day Hospital admission	20 days after discharge	40 days after discharge
T Bil (mg/dL)	3,72	26,44	10,59	2,04	0,55
C Bil (mg/dL)	3,45	24,72	10,27	1,76	0,30
Urea (mg/dL)	59	64	58	26	19
Creatinine (mg/dL)	2,61	1,45	1,08	0,83	0,76
AST (U/L)	2862	472	63	93	23
ALT (U/L)	2929	1945	236	91	22
GGT (U/L)	1160	228	330	201	79
Albumin (g/dL)	3,3	2,4	2,0	1,8	3,72
AP (U/L)	338	159	146	249	88
US-CRP (mg/L)	79,83	7,37	11,3	6,1	0
INR	1,44	2,05	1,13	1,1	1,1
Hemoglobin (g/dL)	11,9	11,6	11,1	8,9	10,6
Leukocyte (/uL)	6170	14900	5450	8540	5750
Atypical lymphocytes (/uL)	0	115	0	0	0
Eosinophils (/uL)	463	581	7	0	167

*AST: aspartate aminotransferase; ALT: alanine aminotransferase; AP: alkaline phosphatase; GGT: *γ*-glutamyl transpeptidase; US-CRP: high-sensitivity C-reactive protein; INR: international normalized ratio; T Bil: total bilirubin; C Bil: conjugated bilirubin.
